# University–Community Partnerships Using a Participatory Action Research Model to Evaluate the Impact of Dance for Health

**DOI:** 10.3390/bs8120113

**Published:** 2018-12-14

**Authors:** Rahshida Atkins, Janet A. Deatrick, Cory Bowman, Ansley Bolick, Ian McCurry, Terri H. Lipman

**Affiliations:** 1 School of Nursing, Rutgers the State University of New Jersey, Camden, NJ 08102, USA; 2 School of Nursing, The University of Pennsylvania, Philadelphia, PA 19104, USA; deatrick@nursing.upenn.edu (J.A.D.); ansleybolick@verizon.net (A.B.); lipman@nursing.upenn.edu (T.H.L.); 3 Netter Center for Community Partnerships, The University of Pennsylvania, Philadelphia, PA 19104, USA; Bowman@upenn.edu; 4 Perelman School of Medicine, The University of Pennsylvania, Philadelphia, PA 19104, USA; Ian.mccurry@pennmedicine.upenn.edu

**Keywords:** physical activity, participatory action research, academic community partnerships, health behavior disparities, outcomes evaluation, qualitative research, content analysis

## Abstract

Little is known about fostering sustainable, collaborative community-academic partnerships that effectively improve physical activity and health in residents of under resourced communities using Participatory Action Research (PAR) driven models. The purpose of this PAR study was to evaluate the impact of an urban, intergenerational, and physical activity dance program by identifying community preferred measurable outcomes that promote program participation and sustainability. A descriptive, qualitative design was employed using semi-structured interview guides to facilitate discussions for two adult focus groups and one youth focus group. Exactly 19 community-residing adults and six youth who lived in urban neighborhoods in West Philadelphia participated in the discussions. The audiotapes were transcribed and analyzed using directed content analysis. Five outcome themes emerged and included: (1). Enhancing the psychological and emotional well-being of the individual, (2). Enhancement of social well-being and management of interpersonal relationships and responsibilities (3). Enhancing and promoting physiologic well-being (4). Changes in health promoting behaviors and skill acquisition, and (5). Concerns about accessibility of dance for health and other physical activity programs in the community. Focused attention to measuring community preferred outcomes can promote sustainability of Dance for Health and possibly other urban-based physical activity dance programs.

## 1. Introduction

Physical inactivity is a major public health concern in the United States (US). Low levels of physical activity among Americans increases the risk of mortality [1,2] and the development of chronic mental and physical health conditions [3] including heart disease, type 2 diabetes [4], obesity [5], hypertension [6], and depression [7]. These chronic conditions cost the US economy billions of dollars each year [8,9,10,11]. Despite these data, nationally nearly 50% of adults [12] and over 70% of youth fail to meet government recommendations for the levels of moderate intensity aerobic activity that is known to enhance mental and physical health (e.g., adult > 150 min a week; youth 60 min/day) [13,14,15].

Physical activity levels are found to be even lower among residents of under-resourced urban communities [16,17], among those who live in poverty, and among those of ethnic minority status [12]. These residents often contend with environmental barriers to physical activity participation such as unsafe neighborhoods and limited access to spaces to participate in physical activity [16,17]. Community partnerships that bring health researchers and community organizations together are proving to be effective ways to improve population health by promoting healthy behaviors such as physical activity participation in these residents [18]. Over the last 20 years, researchers have partnered with community-based organizations to develop and test numerous interventions to promote physical activity participation in low-income, disabled, and ethnic minority residents and residents of under-resourced communities [19,20,21,22]. While it is known that such partnerships have resulted in the creation of physical activity programs that have promoted short-term increases in physical activity participation among these residents, physical activity program attendance and sustainability remains problematic [23]. Rigorous application of community based participatory to program evaluation are needed to ensure that existing programs are continuously refined and tailored to meet the interests, needs, and priorities of the community participants [24,25,26]. These collaborative efforts at program refinement can encourage regular program attendance and program sustainability [25,27].

West Philadelphia is an urban community where 80% of children and about 50% of adults do not meet the US physical activity recommendations and 35% of children do not participate in physical activity even once per week [28,29]. These statistics are not surprising since West Philadelphia is the 8th poorest congressional district in the country (51% live below the poverty line) and has a population that is largely African American [30]. Subsequently, in parts of West Philadelphia, the percentage of adults and youth who are obese (e.g., 39.6%; 22.9%, respectively) is greater than the national average (e.g., 33.0%; 16.6%, respectively). Rates of obesity among Non-Hispanic Blacks and Hispanics are even higher (e.g., 40.1%; 22.9%, respectively) [31]. Decreasing health and health behavior disparities in neighborhoods like West Philadelphia is a government priority [32].

To address these disparities, a free, community based, intergenerational, culturally appropriate physical activity dance program titled Dance for Health (DFH) was developed in West Philadelphia [32]. This program was developed as a partnership between an academic institution and West Philadelphia children and families using a Community Based Participatory Research (CBPR) Approach [24,33]. This University–Academic partnership allowed for collaboration and equitable decision making so that community needs were incorporated into the design of the DFH program [33]. The program was a success and demonstrated sustainability [33]. The program was initially held in a community center and was led by Nurse Practitioner (NP) students in partnership with community high school students. The dance program ran two hours weekly for four weeks in the spring and four weeks in the fall for five years. Over 521 community members (n = 372 adults, n = 149 children) who ranged in age from 8 years to 73 years (m = 35 years) participated during the five years that the program ran [34]. The number of participants at each dance class fluctuated throughout the duration of the program. Most of the participants were adults (71.4%, 372/521; mean age adults = 52.4 ± 14.5) and most of the adult and youth participants were female (adult females = 92.1%, 343/372; youth females = 70.1%, 104/149). All participants self-identified as African American [34]. After the student-led classes ceased, 23 of 26 adults (88%) who attended the last student-run DFH event attended the next community-led DFH event when no students were present [33]. Presently, community led group dance classes continue to operate year-round in multiple locations throughout the community. A complete description of the original program, participant demographics, and its implementation can be found elsewhere [34]. 

Although this program demonstrated sustainability, continued success will depend on rigorous application of CBPR methods that dictate the need to continuously evaluate its impact on community residents to identify factors that contribute to program effectiveness [24,35]. One factor that is proposed to influence the long-term maintenance of physical activity is personal goals and outcome expectations [36,37]. According to many theorists, when the expected outcomes are achieved, desired, realistic, measurable, and bring self-satisfaction to the individual, the behavior producing the outcome is maintained [35,38,39]. Therefore, achievable and realistic outcomes should be defined by community members and should become the focus for measurement when evaluating physical activity promoting interventions [40]. The achievement of desired outcomes also helps researchers to evaluate the impact of health promotion interventions on targeted clients from the client’s perspective. 

To evaluate the impact of the DFH program, this project will utilize a Participatory Action Research (PAR) [41] approach to focus attention on identifying measurable outcomes that are of interest and priority to the community participants who attended. This approach emphasizes two-way communication, collaboration, and equitable participation in the research process [42]. Researcher defined yearly outcomes related to weight, intensity (e.g., mid activity heart rate, pedometer steps, and the BORG rating of perceived exertion scale) [43], attendance, and enjoyment were measured for the DFH participants. However, during a recent program evaluation of outcomes and project sustainability, participants who continue to attend indicated additional outcomes, stating that the program was “good exercise,” “fun,” “safe,” and made them “feel good [33].” These participants responded, however, to a researcher developed sustainability survey [33]. In this study, direct open discussions to determine participants’ priorities for measurable outcomes took place. Other researchers who have used participatory research methods have similarly emphasized outcome measures associated with physical health and physical activity behavior including number of steps, body mass index (height weight), eating habits, blood pressure, and fitness levels [19]. These researchers, however, did not specifically indicate in their reports whether the community members were engaged in identifying the outcomes to be measured [19].

Little is known about fostering sustainable collaborative community–academic partnerships that effectively improve physical activity and health in African American families and residents of under resourced communities, and there are inadequate PAR driven-driven models. This study’s application of a PAR approach to evaluation will serve as a model of facilitation for the identification of the goals, interests, priorities, and preferred outcomes of community members [41,44]. The purpose of this study is to utilize a PAR approach to: (1) Determine the measurable outcomes of interest and priority of the community residents who attend the DFH program and (2): Determine the categories of outcomes that are most preferred by the DFH participants. 

## 2. Methods

### 2.1. Design 

This study employed a qualitative, descriptive design using a Participatory Action Research framework [41]. Meetings were held with community members to determine the common research and evaluation interests between the university and community partners, namely the dance instructors. These instructors indicated their interest in determining the positive effects of DFH on the lives of those who attended. These instructors also discussed the enthusiasm demonstrated by the participants who currently attend and revealed the current participants’ desires to share the impact of this program with other community residents. Those who currently attend are mostly female and adults [33,34]. It was the participants’ belief that evidence that DFH has a positive impact would increase the participation of others and that parents would be very motivated to engage their children in the program. The instructors and participants desired to explore measures of the impact of DFH on the lives of its participants. Academic researchers and the community dance instructors mutually agreed that conducting focus group discussions directly with participants would be the best course to take for identifying the evaluation interests, goals, and priorities of the participants. Plans for the timing, location, and the format for the focus group discussions were made collaboratively to ensure that a sample representative of those impacted by participation in DFH would be in attendance. The results of the focus group discussions were shared with the community members who agreed with the final themes and participated in the review and preparation of these results for dissemination via publication. 

### 2.2. Sampling 

Purposive snowball and convenience sampling was used to obtain a sample that was representative of the past and present participants of DFH. Focus groups were conducted until data saturation was achieved [45,46]. Participants were eligible if they: (1) Participated in at least one DFH physical activity session and were, (2) Able to read, understand, and speak the English language. 

### 2.3. Procedures and Recruitment

The study was approved by the Institutional Review Board of the University of Pennsylvania. Focus group participation was advertised via verbal announcements that were made during and after dance classes and flyers that were distributed during class and were posted on walls. The community dance instructors also made verbal announcements during dance classes and provided reminders about dates and times for those who agreed to participate. For the convenience of the participants, two adult focus groups were held on two different evenings immediately after the dance classes ended. This allowed opportunity for additional persons who happened to be present that day for dance class to participate. The teen focus group was held in a university conference room as they were accompanied by a university community outreach liaison partner.

Each focus group followed the same procedure. The participants were greeted and written informed consent was obtained from each adult participant and youth under the age of 18 provided assent. Afterwards, a demographic data sheet was completed and refreshments were provided. The discussions were tape recorded and lasted for 45 min to 1 h. At the end, participants received a five-dollar VISA gift card. All focus groups were led by a doctorally prepared facilitator and university student research assistants recorded field notes, observations, and handled logistics. 

### 2.4. Data Collection

**Focus Group Guides.** The focus group interview process took place according to a semi-structured focus group interview guide. The topics and probes for focus group discussions were based on categories of holistic health outcomes that are widely accepted in the nursing literature [40]. This framework was used to plan the study (e.g., semi-structured interview guide), however it did not constrain the study method. These outcomes are measurable client or family states, behaviors, or perceptions that largely emphasize health promotion and risk reduction [47,48]. These outcomes are organized into categories, namely physiologic (weight, blood pressure, labs values), psychosocial (emotions, attitudes, moods), functional (activities of daily living), behavioral (regular physical activity), cognitive (knowledge, thinking, learning), home functioning (family support), safety (noise free environment, crime), symptom control (pain, withdrawal), goal attainment (accomplishment of program objectives), satisfaction (enjoyment, contentment, service), and cost outcomes (cost effectiveness) [40]. Since expected outcomes involve an evaluation of the costs and benefits of participation in a health practice and participation is facilitated by goal setting [35,36,39,40], the focus group guides were also developed using this language. 

For example, participants were asked:

1. “What benefits have you obtained from attending this program and what negative things occurred?”
2. “What goals will dance for health help you achieve?”
3. “What do you want us to keep track of or measure to help you measure whether you are getting closer to your goals?”
4. “What social, mental, physical, or thinking outcomes are important to you?”

The questions were structured according to a framework that would encourage the revelation of multiple domains of outcomes. The parent and teen focus group guides were similar, with modifications for language development and educational level. For example, instead of saying bone or muscle “mass,” the term bone and muscle “building” was used. Instead of the term “fat distribution,” the term “fat around your body” was substituted. Two written vignettes were also developed to stimulate thinking about outcomes experienced in participants’ day to day lives. For the parent groups, the subject of the vignette concerned an overweight couple, while for the teen group, the subjects were overweight teen siblings. The discussion proceeded from a general discussion of outcomes, goals, and benefits of DFH and then proceeded to a discussion of more specific outcomes.

### 2.5. Trustworthiness

Trustworthiness was ensured via data triangulation and prolonged engagement [49]. Prior to data collection, triangulation of data occurred via participant observation that allowed for making systematic comparisons and cross-checks concerning the consistency of information that was derived from the interview with what was observed [46]. The focus group leader and two research assistants attended and participated in the two dance classes that took place just prior to the commencement of the adult focus groups. This allowed for interaction between program participant and researchers, establishing the trust that was needed to support openness, honesty, and accuracy during discussions [46,50]. Researchers were able to observe the social interactions during dance classes and compare these to the discussions. At this time and during focus group interviews with adults and youth, field notes were also taken in order to make rigorous comparisons of participant behaviors with the interview data.

### 2.6. Data Analysis

The audiotapes were transcribed verbatim by a professional transcription service using Microsoft Word and then were uploaded into Atlas.ti © after the accuracy was verified. A directed approach to content analysis [51] was used to analyze the data based on the existing nursing frameworks and empirical research. Both inductive [52] and deductive [53] approaches were used to analyze the data with attention to a systematic process that strengthened the rigor of the analysis and the trustworthiness of the data [49,50]. 

The three transcripts were analyzed by two investigators with doctoral degrees, one masters prepared professional, and a trained undergraduate research assistant. All coders were experienced with community engagement research and engagement with residents from under-resourced communities. Coders met frequently by phone and in person. For primary coding of the data, coders read each transcript from beginning to end and coded the data based on start codes that deductively derived from the focus group guide questions and new codes that inductively derived from the data. Subsequent analysis took place via secondary coding where coders identified subcategories for broader codes and collapsed similar codes into an either predetermined category from the primary codes or new single category. For example, the theme chronic health conditions was expanded to distinguish between measures that target “management” verses “prevention” of chronic health conditions. Outcomes related to the measurement of stress and depression were collapsed into one “Mental Health” category. Definitions and descriptions for each code were written, revised, and re-written as the agreement upon themes were finalized. The coders were trained and supervised by a senior investigator who also agreed with the final themes. 

## 3. Results

Exactly 25 persons participated in three separate focus groups (two adult groups and one youth group). There were seven adults in the first focus group and 12 in the second who ranged in age from 43 to 71 years (M = 55.85; SD = 8.55). All adults were female (N = 19; 100%) and identified as Black/African American. There were six teens in the youth focus group who ranged in age from 17 to 19 years (M = 17.50; SD = 0.84). Four youth identified as Black/African American, two as Hispanic/Latino, and one as biracial (White and Black/African American); five youth were female and one was male. Overall, the participants in this study were mostly adults (76%; 19/25) and female (96%; 24/25). The youth (24%; 6/25) were also almost all female youth (83%; 5/6) and all middle to late adolescents (100%). In terms of health, one female youth indicated a medical history of asthma. Adults indicated a history of arthritis (n = 5), high blood pressure (n = 7), diabetes (n = 3), high cholesterol (n = 1), sleep apnea (n = 1), asthma (n = 1), stroke (n = 1), heart disease (n = 1), cancer (n = 1), and gallstones (n = 1). Some adults indicated more than one condition. Additional demographics for participants are found in Table 1 and Table 2. 

Participants revealed personal goals, positive (benefits) and negative outcomes, and barriers and facilitators of participating in DFH. Five broad themes emerged representing the priority outcomes that the community prefers to measure and concerns that surfaced because of their participation in DFH. Saturation was reached with just two adult and one youth group since they expressed identical themes. The youth and parent transcripts mainly offered similar information and saturation was reached utilizing the three groups making most of the final themes’ sentiments of both age groups. These themes and outcomes will be discussed along with quotations from members of the three focus groups. 

Theme 1: Enhancing the psychological and emotional well-being of the individual.

### 3.1. Enhancing Mental Health 

Both youth and adults reflected on outcomes associated with changes in their mental health. They discussed the impact of DFH on their emotions, moods, and cognitive abilities. DFH provided a social atmosphere that supported them cognitively and emotionally. Improvements in these outcomes were set as pre-determined goals and occurred unexpectedly because of attendance and encouraged continued attendance. Subsequently, both adults and youth specifically mentioned the desire to measure depression levels, other emotions, and memory, while adults additionally mentioned anger, stress, and anxiety. When directly asked what they would like to measure, adults stated, “depression…the mood part of it…“Anger?” “I would say…stuff like anxiety and depression,” “memory,” and “my focus.” Youth specifically mentioned wanting to measure “intellect,” “emotionally…yeah, both of them.”

**Adult:** “It be good to measure stress levels before and after the class.”

**Youth:** “You should measure all different aspects...one day like your usually like a physical person, but since there are not happy or anything, they get depressed and stop dancing and stop showing all this stuff they used to do.”

### 3.2. Increasing Self-Confidence 

Both youth and adults reflected on outcomes associated with changes in their levels of confidence. They discussed the impact of dance for health on their emotions and attitudes toward the self. The encouragement they received from others while participating in DFH provided a sense of achievement, boosts in self-esteem, and immediate gratification to their focus and efforts in learning to dance. Improvement in these outcomes occurred unexpectedly and thereby encouraged continued attendance. Subsequently, they desired to measure these variables. When asked directly what they would like to measure, adults stated, “So definitely a confidence measure,” and “Self-esteem? Oh yes.” Youth mentioned specifically wanting to measure “self-consciousness” and “body image.”

**Adult:** “And I just wanted to add like when you come like, you know, to exercise and dance and stuff, it’s like, um a confidence booster.”

**Youth:** “So like remembering the dances, remembering the people that’s there will help you like probably remember like, oh, I got this.”

### 3.3. Fun and Enjoyment 

Both youth and adults reflected on how fun DFH was and the enjoyment it brought to their lives. Fun was discussed as a consequence of attending and as a motivating factor for attending. Participants enjoyed the music, dancing, and sense of community and identified DFH as one of their main sources of recreation and relaxation throughout the week. Adults and youth directly stated that they would like to measure “fun” and “enjoyment.”

**Adult:** “But when you come here, that’s why it’s so good…not only that it’s fun…cause I always tell my class it’s fun and fitness all in one.”

**Youth:** “That was important that they ask them see If they enjoyed it...cause some programs…they don’t really ask your opinion about it…“I’m able to have fun with like my peers…and listen to some good music.”

Theme 2: Enhancement of social well-being and management of interpersonal relationships and responsibilities.

### 3.4. Enhancing Socializing 

Both youth and adults reflected on outcomes associated with social functioning. They discussed the impact of DFH on socializing, perceived social support, culture, and social networking. As reported, the acceptable intergenerational social atmosphere enhanced perceived social support and encouraged regular attendance. The network of relationships that the participants developed within the program and spending time with close friends and family members in a fun and constructive intergenerational environment was a major factor in their continued attendance and participation in DFH. This atmosphere was generally enjoyed by most except for one male youth who recommended separating adults and youth. He saw the intergenerational aspect as a barrier since his taste in music and type of dance differed from the adults. He stated, “…I think we should have like a separate class...like younger people like doing dances that we know of…Because...they wasn’t really doing dances that we knew of…they was playing stuff like from the eighties…like cupid…shuffle, the electric slide.” Another female youth commented against separation by age, but recommended playing music that is appealing to all age groups. She stated, “not a separate class, but a general mix of the young and elders, …kind of mixed up the music…I’m not a big fan of younger dances...my little brother...loves older music than the new music...he only know a couple new dances from within like the four years.” Subsequently, both adults and youth desired to measure social outcomes. Adults specifically mentioned wanting to measure, “Yeah social.” Youth wanted to measure “networking” and “socially,” “motivation,” and “cultural beliefs.”

**Adult:** “In the beginning I was supporting this program called investing in ourselves, but because of the bonding and the camaraderie and the meeting people, this is now a mandatory obligation…it is now a commitment…now just come here.”

**Youth:** “It’s a good social setting which brings together the young and the older folks...I was able to meet great, um, seniors and talk to them about health and how important it is to stay healthy.”

### 3.5. Assistance with and Relief from Caregiving 

Adults reflected on outcomes associated with their social roles. As explained by participants, the reduction in caregiver stress and caregiving relief provided by DFH were important motivating factors for continued attendance. Attendance at DFH was a way to refocus attention on themselves after years of caregiving for children and aging parents and/or as a way to manage their caregiving roles and responsibilities. DFH provided assistance with and escape from caregiving. Youth did not discuss caregiving.

**Adult:** “And she’s eighty-nine, and she just likes to get out, …since...I’m home with her now taking care of here, you know, wherever I go, she goes…for me and my mom its bringing us closer…we have something to do together.”

Theme 3: Enhancing and promoting physiologic well-being. 

### 3.6. Management of Chronic Health Conditions

Adults reflected on outcomes associated with the management of chronic health conditions that either they or their participating family members were diagnosed with. Members often named the chronic health condition that prompted them to attend DFH and stated how the DFH program helped with managing the condition. Adults mentioned having diabetes, hypertension, bursitis, overweight/obesity, bunions, and arthritis. Some of their goals for attending dance were health targeted and regarded the conditions above as many desired to lose weight, lower their blood pressure, discontinue or decrease the use of prescribed medications, and limit their pain levels. Adults specifically wanted to measure blood pressure, heart rate, BMI, height, weight, waistline, diabetes measures, calories burned, cardiovascular stimulation, medication changes, and aches/pain levels before and after exercising. Youth did not mention managing chronic conditions.

**Adult:** “So I came to improve my health cause you know I’m diabetic and I have other, you know health issues…I lost sixty pounds last year and kept it off.”

### 3.7. Prevention of Chronic Health Conditions

Both youth and adults reflected on outcomes associated with prevention of chronic health conditions, which emerged as a theme that was separate from management of health conditions. Adults discussed preventing chronic health conditions such as diabetes, high blood pressure, and Alzheimer’s disease. When asked to specifically state what they would like to measure, adults’ statements included, “weight loss,” “blood pressure?,” and “focus.” Youth made general statements about “staying healthy” and avoiding “medication.” Youth specifically mentioned “weight loss,” “calorie burnage,” and “blood pressure.”

**Adult:** “It’s good because it helps the heart rate, and it’s like the dance helps the heart rate, and I never had high blood pressure or sugar diabetes or anything, but I have to say that by me dancing...”

**Youth:** “I guess just getting more of an exercise way and try not to be on pills and medication for my stuff…You know, try a different approach.”

### 3.8. Enhancing Mobility 

Adults reflected on outcomes associated with Mobility. Adults suffering from chronic musculoskeletal conditions frequently commented on the impact of DFH on their physical mobility. DFH allowed participants to keep mobile and physically flexible more often than if they were to follow their normal routine.

**Adult:** “It keeps you limber. If you come all year round, it keeps you limber even in the wintertime when your bones get stiff. When you start moving you feel better.”

Theme 4: Changes in health promoting behaviors and skill acquisition. 

Both youth and adults reflected on outcomes associated with promoting healthy behaviors and skill acquisition. Participation in DFH helped them to engage in positive health behaviors and improve interpersonal skills and behaviors. Adults discussed how attendance at DFH encouraged them to increase their activity levels when they were not at DFH. Youth discussed becoming conscious of their eating habits, sedentary behaviors such as cell phone use, and activity levels such as number of steps taken. Youth spoke about moving and being active as a way to stay healthy and commented on the adults’ mobility, expressing surprise with how mobile the adults were. Adults and youth wanted to measure their “steps.” Youth additionally wanted to measure “common courtesy,” “time,” “prioritizing,” and “leadership.”

**Adult:** “I kept that pedometer on myself, trying to achieve the ten thousand and fifteen thousand steps…so…It encourages you to, like they say, keep active, and its free, and I feel like I can dance finally.”

**Youth:** “I would want to measure what I eat…Like how many steps you are taking, …and only take a break from my phone three times…that’s a goal…I was active like the whole…I want to try to stay active?”

Theme 5: Concerns about Accessibility of dance for health and other physical activity programs in the community. 

### 3.9. Programs in the Community

Both youth and adults reflected on accessibility concerns that surfaced as a result of participation in DFH. These included the ease or difficulty with attendance and facilitators and barriers to attendance. Attendance was taken and is often a goal for measuring the success of physical activity programs. The lack of cost of DFH facilitated attendance. Adults reported potential barriers such as issues with transportation, safety, parking, and timing. Though difficult, these issues did not prevent them from attending DFH because they enjoyed the program more. Youth also named other convenient exercises and physical activity programs that were in walking distance and that they attempted to participate in regularly.

**Adult comment:** “60th and spruce has been on the news…we, for…most part, leave in a group formation…and when it’s dark at 5:00, I’m concerned for my safety so…still come here because I like it overrides my paranoia about getting back safely.”

**Youth comment:** “I walk basically everywhere…“I would try to get more time at the gym as much as I could, I would probably participate in sports as much as I could.”

Additional statements for adults and youth can be found in Table 3 and Table 4 below.

## 4. Discussion

The health impact of DFH was broad, as indicated by the five themes that emerged, and suggested that both adults and youth preferred to measure a variety of holistic health domains [40]. Of note is that most of the preferred outcome themes represented psychosocial and emotional categories of health such as mental health, self-confidence, socializing, fun, and caregiving assistance. These findings underscore the need to give critical attention to the social determinants of health behaviors and those “upstream” factors that influence sustained participation in physical activity programs [54]. The current program measures a number of the outcomes that were identified by the community; cardiovascular health, enjoyment, and whether DFH results in incorporating dance at home. The participants identified additional outcomes of interest that would add to the benefit of the program. Many admitted to initially attended to achieve personal physical health-related goals. However, these participants found a positive social environment that created a sense of connectedness and safety and gave them confidence to engage in dance for physical activity regularly and to explore other personal and healthy behaviors to improve overall physical and mental health. Social determinants were important aspects of continued physical activity dance participation for these residents who attended. It must be noted, however, that those who agreed to participate in this study were mostly middle aged to older adults (76%; 19/25) and females (96%; 24/25). This gender and age composition closely represents those who primarily attended the DFH program when it was student-led [33,34]. As indicated by a comment from a male youth above who discussed the intergenerational aspect as a potential barrier, perhaps the program has elements that are not appealing to males, younger adults, and/or youth. 

These findings are consistent with previous research that examines outcomes of general physical activity promoting interventions. Psychosocial and emotional outcomes of physical activity that are consistent with the emerging themes of self-confidence (exercise self-efficacy, self-esteem, competence, confidence) [55,56] and socializing (social support) [57,58] and feelings of enjoyment [59,60] have often improved after interventions to promote physical activity were employed in disadvantaged populations [16,61,62]. Perceiving social support or lack there-of [16,63], social connections [59], and family support [60] in community settings [64] often facilitates physical activity maintenance in African American women. Evidence reveals that family based physical activities provide the social support that is necessary for promoting active lifestyles for adults and youth [61,65]. In African American women, higher levels of social support and exercise self-efficacy has been shown to predict physical activity participation [66,67]. Caretaker roles and responsibilities (i.e., lack of childcare, caring for extended family) [16] are often cited as barriers to physical activity participation for African American women [65]. The current findings provide new knowledge about the positive psychosocial impact of physical activity group-dance in a sample of mostly middle-aged to older adults and middle to late adolescent youth who prefer to measure these psychosocial outcomes. 

Psychosocial outcomes consistent with the theme, Mental Health, such as depressive symptoms [68,69,70] and perceived stress [70,71], have also improved during interventions to promote physical activity in disadvantaged populations. Exercise reduced depressive symptoms and stress levels in disadvantaged women [71]. High levels of depressive symptoms are often inversely associated with general physical activity in diverse ethnic groups of women [72], including African American women [73] and in youth [74,75]. High levels of stress are inversely associated with physical activity participation in women [76] and youth [77]. Many participants reported that DFH helped with memory, focus, and intellect and, thus, participation in group dance for physical activity may be an effective strategy to manage and/or prevent mental health problems in persons with similar demographics. This study provides new knowledge regarding the positive mental health benefits of group-dance for physical activity in residents with similar demographics to the current sample. These residents subsequently prefer to measure these aspects of mental health that have improved as a result of their participation in DFH.

Measures of physiologic health associated with managing chronic health conditions were the second most frequently mentioned outcomes by adults. Physiologic measures such as height, weight, and blood pressure are commonly measured outcomes in studies that target African American women [78,79,80]. These outcomes reflected pre-set health related goals for attending the DFH program. Health promotion begins with goal-setting [36] that often facilitates physical activity initiation [65] and maintenance [63] in African American women. Height, weight, and heart rate were measured, however adults also wanted measures of symptom control (e.g., pain management, diabetic control). Symptom control is rarely measured as an outcome for physical activity in non-clinical community samples of adults. This study reveals the benefits of group-dance on symptom control in a sample of community dwelling adults who also prefer to measure these as an outcome of physical activity group-dance participation. 

Behavioral outcomes, such as decreased television and computer use and increasing physical activity levels, were the second most common outcome category that was mentioned by youth and are commonly reported for interventions that are designed to promote general physical activity in youth [61]. Personal goal-setting for specific health promoting behaviors, such as healthy eating [61], is frequently a component of successful physical activity promoting interventions in youth. This study reveals that youth desired to measure outcomes associated with changes in sedentary and health promoting behaviors, which are factors that are not currently being measured as a component of the DFH program. This study also provides new knowledge regarding the positive impact of group-dance for physical activity on youth’s consciousness for decreasing sedentary behaviors and increasing physical activity levels. 

Issues of accessibility such as costs and safety were also discussed, however they were not always specifically reported as desired measurable outcomes by participants. Unsafe neighborhoods [60,81] and costs [72,82] are often cited as barriers to physical activity participation for African American women [65]. These barriers were offset by the positive components of DFH that facilitated attendance. 

### Limitations/Strengths

A purposeful, convenience sample for this study is inherently biased due to subject self-selection of participants and the limited size of the sample compared to those who participated for the duration of the DFH program. The sentiments of adult males are absent from this analysis. The expressions represent the sentiments of mostly middle aged to older adult females and some youth (e.g., late adolescents) and may not reflect the sentiments of all who attended the DFH program over the five-year period, such as adult males, younger adults, younger children, and early adolescents [33]. However, the age and gender composition of this sample closely reflects those who have primarily participated in DFH. 

## 5. Conclusions/Implications

The achievement of goals and the continuous experience of positive expected outcomes contributes to the sustainability of DFH. Personal goals provide incentives and guidance for engagement in health promoting habits [36] and when measurable outcomes are achieved, the sense of satisfaction encourages long-term engagement [36]. Use of a PAR approach ensured that participants determined the purpose and directed the focus of this study towards identifying the outcomes of priority and interest to them. Their statements indicate that the DFH program provides an environment that supports them to maintain a physically active lifestyle by supporting their utilization of personal and social resources as assets to maintain regular attendance for physical activity participation [83]. 

To promote sustainability of the DFH program, outcome measures that are consistent with the goals and expectations preferred by the DFH participants should be added. Adding measures of perceived social support [84,85], confidence [86], and caregiver strain [87] will more thoroughly assist with evaluating the overall positive impact of the DFH on these community participants and enhance sustainability. Measures of satisfaction to assess enjoyment should continue to be administered as well. Adding measures of mental status such as cognitive functioning [88], depressive symptoms [89,90,91,92,93], stress [94], anger [95], and anxiety [91] to our intergenerational program will ensure a more thorough evaluation of the overall impact of DFH on the mental health of all participants. A satellite DFH program with participants who are elders experiencing memory problems evaluates the impact of DFH on anxiety and memory [96]. Additional personal physiologic outcome measures such as pain questionnaires [62,97,98] or biochemical markers of chronic disease (e.g., Hgb A1C) [23] should also be added. Measures of mobility are not commonly measured as outcomes of physical activity participation in non-clinical samples [26,87,99]. These measures can also be added since these were positive outcomes that were reported by the DFH participants. Youth may be motivated to continue if personal goal setting as a formal component of the DFH program can be instituted, such as tracking dietary changes, decreases in calorie intake, and sedentary behaviors. This tracking will additionally allow for self-evaluation of the overall impact of DFH on healthy behaviors. Personal feedback detailing achieved outcomes may also increase motivation for participation. As one participant stated, “since you keep a record every week, you know, measuring our weight and everything, why don’t you at a certain time period tell us…give us some sort of a report card.” Since this report was written, a health summary for participants has been added to the program based on this recommendation. 

Future research should determine the types of social support and network support pathways that fostered sustained participation in DFH for these participants. This knowledge will allow researchers to collaborate with community participants to ensure that program adjustments will incorporate innovative ways to facilitate these specific support pathways. In addition, given that most participants who have attended the DFH group-dance events and those who continue to be engaged are mostly middle aged to older adults and females [33,34], future research should also explore strategies to brand participation in DFH events as gender neutral. This research should also explore strategies to make this sustainable physical activity program more appealing to young adults, younger children, and early adolescents.

This new knowledge gained will be used to refine the DFH program and, consequently, its sustainability. This program can eventually serve as a model for promoting the sustainability of physical activity dance programs for select residents of under-resourced communities since community-based PAR approaches are known to facilitate partnerships that increase physical activity participation and program sustainability [100,101,102]. This evaluative process can also inform the development and collaboration of additional University—Community partnerships in efforts to develop new and refine existing programs that promote physical activity maintenance in residents of under-resourced communities [12,103,104]. 

## Figures and Tables

**Table 1:** Frequency Distribution of Selected Demographic Variables Youth.

Characteristics	N = 6	Percentage (%)
Age in years		
Mean Age	17.50 (SD = 0.84)	
Range	17–19	
17	4	66.7
18	1	16.7
19	1	16.7
Gender		
Female	5	83.3
Male	1	16.7
Ethnicity/origin (or Race)		
Black or African American	3	50.0
Hispanic or Latino	2	33.3
White and Black	1	16.7
Grade		
Grades 9 through 11	3	50.0
Grades 12 or GED	3	50.0
Neighborhood		
West Philadelphia, PA	3	50.0
North Philadelphia, PA	2	33.3
North/West Philadelphia, PA	1	16.7

GED = General Educational Development; PA = Pennsylvania.

**Table 2:** Frequency Distribution of Selected Demographic Variables Adult.

Characteristics	N = 19	Percentage (%)
Age in years		
Mean Age	55.84 (SD = 8.55)	
Range	43–71	
40 to 49	4	21.1
50 to 59	9	47.4
60 to 69	5	26.3
70 to 79	1	5.3
Gender		
Female	19	100
Male	0	
Ethnicity/origin (or Race)		
Black or African American	17	100
No answer	2	
Marital Status		
Divorced	7	36.8
Never Been Married	9	47.4
Married	1	5.3
Separated	1	5.3
Widowed	1	5.3
Highest Grade Completed		
Grade 12 or GED	3	15.8
College 1 to 3 years	12	63.2
College 4 years	2	10.5
Graduate School	2	10.5
Neighborhood		
West Philadelphia, PA	12	63.2
Darby, PA	2	10.5
North Philadelphia, PA	1	5.3
North West Philadelphia, PA	1	5.3
Yeadon, Delaware County PA	1	5.3
No Answer	2	10.5
Employment Status		
Employed for Wages	12	63.2
Retired	3	15.8
Self-Employed	1	5.3
Unemployed	1	5.3
Disabled	2	10.5
Yearly Income		
$10,000 to $19,999	1	5.3
$20,000 to $39,999	6	31.6
More than $40,000	5	26.3
No Answer	7	36.8

GED = General Education Development; PA = Pennsylvania.

**Table 3:** Themes and Quotations: (N = 25).

Themes	Sub-Themes	Quotations
**Theme 1:** Enhancing the psychological and emotional well-being of the individual	Enhancing mental health	**Adult:** “If you are a depressed person and you have social issues and all of that, it cannot happen in this class...this class will make you talk to people.” **Youth:** I think the survey thing would be a good thing to do so that we would know how the person feel…So I think all the things.”
	Increasing self-confidence	**Adult:** “Yeah, you do get confidence in yourself...It makes you feel good about yourself, you know.” **Youth:** You may want to ask people if they feel okay with their bod, right…body image.”
	Fun and enjoyment	**Adult:** “Not only that it’s fun…it’s fun and fitness all in one.” **Youth:** “Dance for Health was fun.”
**Theme 2:** Enhancement of social well-being and management of interpersonal relationships and responsibilities	Enhancing socialization	**Adult:** “It helps me socialize and do something besides go to work all week.” **Youth:** “I enjoyed Dance for Health because like I met people who like to dance almost as much as I do...To have fun, learn something new, and meet other people.”
	Assistance with and relief from caregiving	**Adult:** “So I’m in a…rebirth…I have to figure out how to do things for me... so I started coming…My son is grown now and I don’t have any…things to do, so it helps me socialized…do something for yourself.”
**Theme 3:** Enhancing and promoting physiologic well-being	Management of chronic health conditions	**Adult:** “Pain management, Yeah, the pain…Put the pain down.”
	Prevention of chronic health conditions	**Adult:** “Staying healthy…I’ll go with that…So it kind of makes me more determined to remain focused…I hear when you stop focusing, Alzheimer’s sets in, and I just refuse to lose to that because I didn’t evaluate and measure my focus.” **Youth:** “I’m saying like you get younger people in it, like promote healthy habits.”
	Enhancing mobility	**Adult:** “Cause I normally wear like a brace on my knee…I haven’t had to use that.”

**Table 4:** Themes and Quotations: (N = 25).

Themes	Quotations
**Theme 4:** Changes in health promoting behaviors and skill acquisition	**Adult:** “But another thing I’ve done is our pedometers we were given, we used it even after…You know, when we get ready to go to work or we’re walking somewhere, we put it on.” **Youth:** “I had a goal was to eat more healthy…learn something new…like make sure I dance and stuff because that would be my exercise for the day.”
**Theme 5:** Concerns about Accessibility of dance for health and other physical activity programs in the community	**Adult:** “Like they say, keep active and it’s free…and that’s great…I tried a couple of other line-dancing…none of that…you just go in and they just start…They don’t, you know, tell you the steps.” **Youth:** “Go to the gym together.”
